# Carbon ion radiotherapy for inoperable pediatric osteosarcoma

**DOI:** 10.18632/oncotarget.25165

**Published:** 2018-05-01

**Authors:** Osama Mohamad, Reiko Imai, Tadashi Kamada, Yuki Nitta, Nobuhito Araki, the Working Group for Bone and Soft Tissue Sarcoma

**Affiliations:** ^1^ Research Center Hospital for Charged Particle Therapy, National Institute of Radiological Sciences, National Institutes for Quantum and Radiological Science and Technology, Chiba, Japan; ^2^ University of Texas Southwestern Medical Center, Department of Radiation Oncology, Dallas, Texas, USA; ^3^ Ashiya Municipal Hospital, 3 9-1 Asahigaoka, Ashiya City, Hyogo, Japan

**Keywords:** carbon ion, radiotherapy, charged particles, pediatric, osteosarcoma

## Abstract

**Background:**

Unresectable pediatric osteosarcoma has poor outcomes with conventional treatments.

**Results:**

Twenty-six patients aged 11–20 years (median 16) had inoperable osteosarcoma of the trunk (24 pelvic, 1 mediastinal and 1 paravertebral) without any other lesion at initial examination. There were 22 primary, 1 locally recurrent and 3 metastatic cases. Median CIRT dose was 70.4 Gy RBE (relative biological effectiveness) delivered in 16 fractions. Median follow-up was 32.7 months. Overall survival was 50.0% and 41.7% at 3 and 5 years, respectively. Ten patients survived for more than 5 years (range 5–20.7 years). Local control was 69.9% and 62.9% at 3 and 5 years, respectively and progression-free survival was 34.6% at 3 and 5 years. Only largest tumor diameter correlated with 5-year overall survival and local control. There were 4 grade 3-4 CIRT-related late toxicities, 1 case of bone fracture and no treatment-related mortalities. All patients (except 1) were able to ambulate after CIRT.

**Conclusions:**

CIRT was safe and efficacious in the treatment of inoperable pediatric osteosarcoma with improved local control and overall survival compared to conventional treatments.

**Methods:**

We retrospectively reviewed the records of pediatric and adolescent patients who received carbon ion radiotherapy (CIRT) for inoperable osteosarcoma between 1996 and 2014.

## INTRODUCTION

Osteosarcoma, the most common primary bone malignancy in children and adolescents, is a rare radio-resistant cancer [1]. Neoadjuvant chemotherapy, followed by surgical resection, and further adjuvant chemotherapy is the typical treatment approach for high-grade osteosarcomas [2]. While this approach yields an acceptable overall survival for resectable cases, outcomes remain poor for unresectable osteosarcomas such as those in the pelvis or trunk. Luckily, these cases only account for a fraction of all osteosarcomas [3–5]. Data on the treatment of unresectable osteosarcoma cases in pediatric and adolescent patients is limited. Given the resistance to treatment, the deep location and proximity to radiosensitive organs such as gastrointestinal tract, spinal cord and nerves, novel methods are needed to treat unresectable pelvic/trunk osteosarcomas.

Carbon ion radiotherapy (CIRT) has stronger biological effects and better dose distribution compared to photon- and proton-based therapies [6, 7]. These physical and biological advantages made CIRT an attractive radiation modality for radio-resistant and deep-seated tumors, or tumors in close proximity to sensitive organs such as trunk osteosarcomas. CIRT has been reported to have good outcomes for adult patients with trunk osteosarcoma [8] and other unresectable bone and soft tissue sarcomas [9–12]. However, no prior study has yet reported results for unresectable trunk osteosarcomas in pediatric and adolescent patients. We, hereby, present the first-ever experience using CIRT for the treatment of unresectable trunk osteosarcoma in pediatric and adolescent patients.

## RESULTS

### Patient and treatment characteristics

Between November 1996 and July 2014, 26 pediatric and adolescent patients with unresectable, truncal, and histologically proven osteosarcomas were treated with CIRT. Patient and tumor characteristics are shown in Table 1. The median age at CIRT was 16 years (range 11–20). There were more males (69%) than females. All cases were high-grade osteosarcoma, the majority of which were osteoblastic (50%) followed by chondroblastic (31%) histologies.

**Table 1:** Patient characteristics

Characteristic	^#^patients (%)	Median (range)
Age		16 (11–20)
Sex		
Male	18 (69%)	
Female	8 (31%)	
ECOG Performance status		
1	19 (73%)	
2	7 (27%)	
Pathologic subtype		
Osteoblastic	13 (50%)	
Chondroblastic	8 (31%)	
Fibroblastic	3 (11%)	
Telangiectatic	1 (4%)	
Unknown	1 (4%)	
Tumor status		
Primary	22 (85%)	
Local recurrence (prior surgery)	1 (4%)	
Metastatic	3 (11%)	
Largest tumor extent (cm)		9.25 (6–20)
Prior surgery		
Yes	4 (15%)	
No	22 (85%)	
Prior chemotherapy		
Yes	26 (100%)	
No	0	
Response to chemo		
Partial response	3 (11%)	
Stable disease	9 (35%)	
Progressive disease	9 (35%)	
Other^a^	5 (19%)	

Abbreviations: ^#^number; ECOG, Eastern Cooperative Oncology Group.

^a^Other = unknown response (3) and incomplete chemotherapy regimen (2).

The majority of patients had primary disease (22 patients), 1 had a locally recurrent iliac lesion after initial surgery, and 3 had metastatic disease. The 22 primary cases included: 2 radiation-induced cases (1 sacral, 1 iliac), and 20 de novo pelvic cases (11 sacral, 8 iliac, 1 pubic). The 2 patients with radiation-induced osteosarcoma have received prior pelvic RT for Wilm’s tumor and chronic granulomatosis, respectively. The 3 metastatic cases included: 1 mediastinal mass (upper extremity primary status post resection), 1 chest wall mass (upper extremity primary status post resection), and 1 iliac mass (lower extremity primary status post resection). All patients, including the metastatic cases, did not have any other lesion except the irradiated lesion at our initial examination. Thus, in total, 4 patients received prior surgery including 3 metastatic cases and 1 primary case. All patients started neoadjuvant chemotherapy prior to CIRT but 2 did not finish their full course. Information about response to chemotherapy is available on 21 patients: 3 had partial response, 9 had stable disease and 9 had progressive disease.

**Figure 1:**
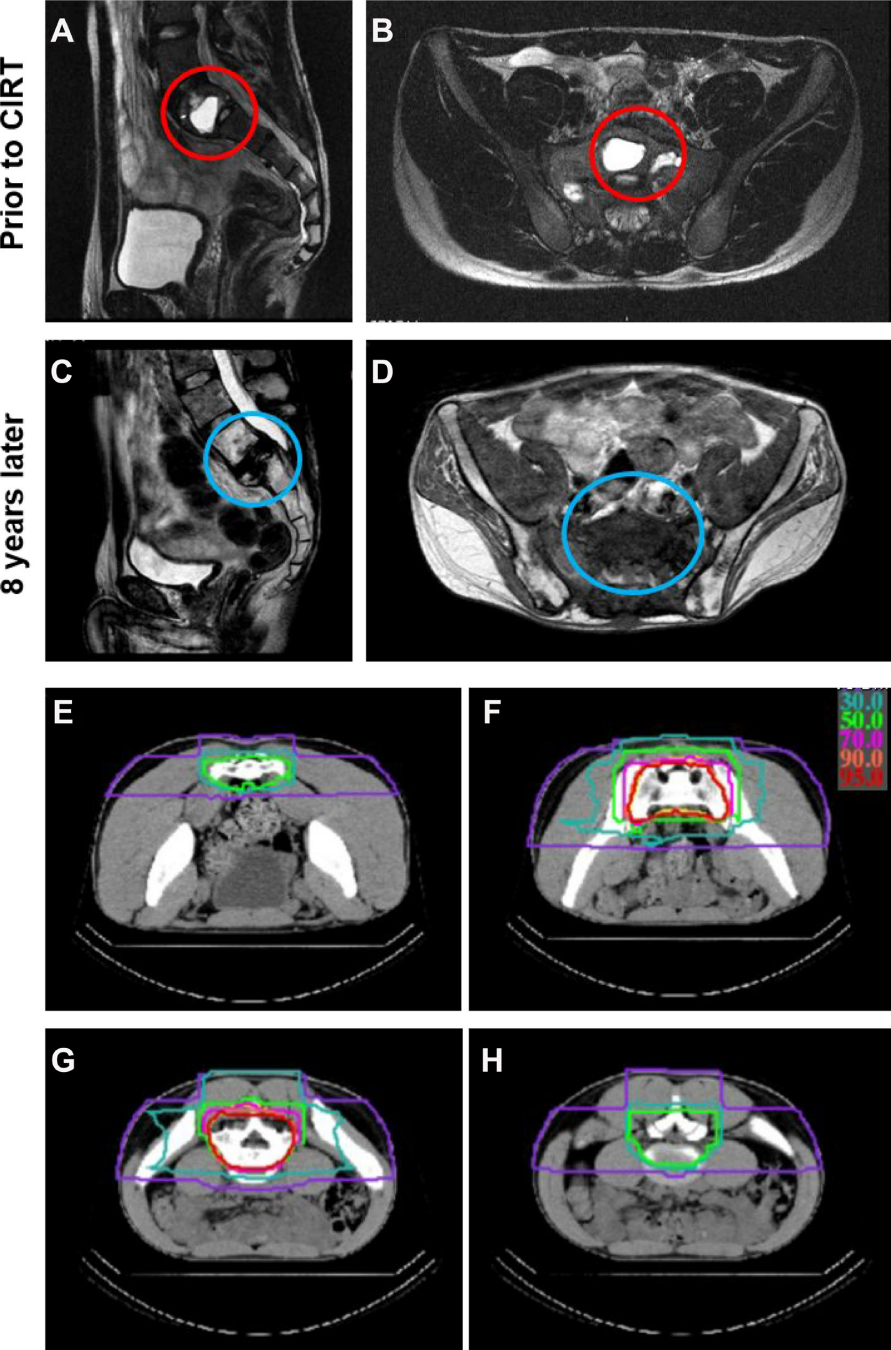
An example of a treated case with unresectable sacral (S1) osteosarcoma (**A**–**B**) show magnetic resonance imaging (MRI) scans prior to carbon ion radiotherapy (CIRT) in the sagittal (A) and horizontal (B) planes with the tumor highlighted in the red circles. (**C**–**D**) show MRI scans 8 years after CIRT in the sagittal (C) and horizontal (D) planes without any tumor recurrence (blue circles) but with obvious fracture in S1. (**E**–**H**) show selected computed tomography (CT) cross sectional views of the CIRT dose distribution (isodose lines: red 95%, orange 90%, pink 70%, light green 50%, green 30%, purple 10%).

Table 2 summarizes the characteristics of CIRT treatments. Twenty-four patients were irradiated to their pelvis (12 sacral, 11 iliac, and 1 pubic). The remaining two patients received CIRT to the mediastinum and chest wall paravertebral area, respectively. The median irradiation volume was 452 cm^3^ (172–1774 cm^3^). The majority of patients received 70.4 (18 patients) or 73.6 (3 patients) Gy RBE in 16 fractions. Figure 1 shows an example of a patient with sacral (S1) osteosarcoma with pre- and post-CIRT magnetic resonance imaging (a-d) and the CIRT treatment plan (e-h).

**Table 2:** Characteristics of carbon-ion radiotherapy treatments

Characteristic	^#^patients (%)	Median (range)
Irradiation site		
Pelvic	24	
Spinal/paravertebral	1	
Mediastinum	1	
Target volume (cm^3^)		452 (172–1774)
Radiation dose, total (Gy RBE)		70.4 (52.8–73.6)
≤64^a^	5	
70.4	18	
73.6	3	
Dose per fraction (Gy RBE)		4.4 (3.3–4.6)

Abbreviations: ^#^number; cm^3^, cubic centimeters; Gy RBE, Gray Relative Biological Effectiveness.

^a^This includes 52.8 Gy RBE (1), 57.6 Gy RBE (1), and 64 Gy RBE (3).

### Treatment outcomes

**Figure 2:**
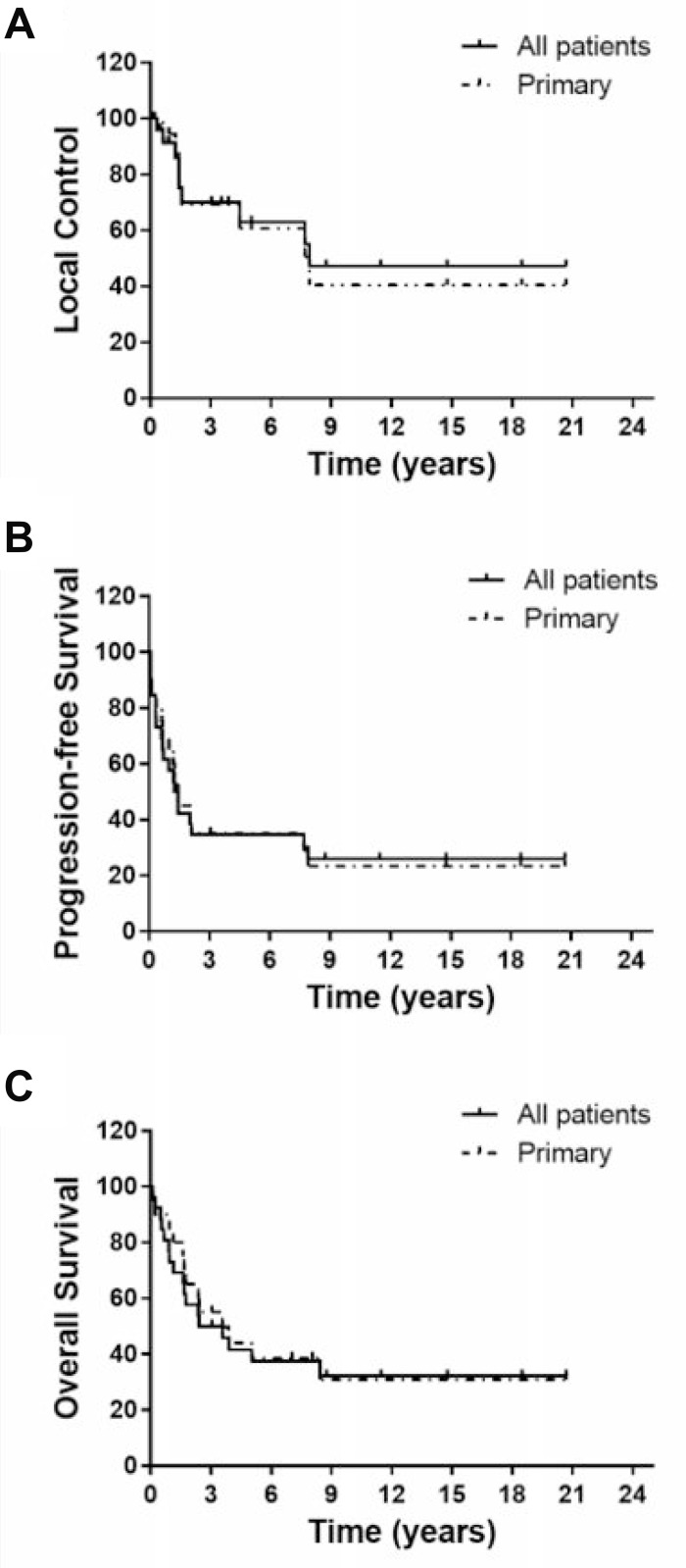
Kaplan–Meier curves of local control (**A**), progression-free survival (**B**) and overall survival (**C**) for all patients together (*n* = 26) or the 20 primary pelvic cases (not including metastatic, locally recurrent or radiation-induced cases).

Median follow up was 32.7 months (1.2–248 months). Nine patients were alive at the end of the study with a median follow up of 137.5 months. Ten patients survived for more than 5 years. Overall survival (OS) was 50.0% and 41.7% at 3 and 5 years, respectively (Figure 2). Median survival was 42.8 months. Local control (LC) was 69.9% and 62.9% at 3 and 5 years, respectively. Median time to recurrence was 17.3 months (3.8–95 months). Late local recurrence (>5 years) occurred in 2 cases (at 95 and 92 months). Fourteen cases developed distant metastasis with a median time to metastasis of 10 months (0.75–94.9 months). First site of metastasis was lung (8 cases), bone (2 cases), and others (gluteus muscles, adrenal, pelvis LN, and pleural effusion; 1 case each). Progression-free survival (PFS) was 34.6% at 3 and 5 years.

Given the known difference in prognosis among true primary, recurrent, radiation-induced and metastatic cases, we analyzed outcomes for the 20 primary pelvic cases separately (not including metastatic (3), locally recurrent after surgery (1) and radiation-induced (2) cases). OS was 55.0% and 44.0% at 3 and 5 years, respectively. LC was 69.3% and 61.0% at 3 and 5 years, respectively. PFS was 35.0% at 3 and 5 years. These numbers are not statistically different compared to the whole cohort (Figure 2 and Table 3). Of the two radiation-induced cases, one died with local recurrence (4 months after CIRT) but no distant metastasis and the other died with distant metastasis (lung, 8 months after CIRT) without local recurrence. The non-metastatic locally recurrent case was still alive at the end of the study (105 months since CIRT) without recurrence or metastasis. None of the 3 metastatic cases had recurrence at the irradiated site. Two, however, had metastasis (1 lung and 1 pleural effusion) and both patients have died. One patient is still alive without recurrence or metastasis (137 months since CIRT).

**Table 3:** Univariate analysis of the 5-year local control and overall survival

	^#^patients	5-year OS, %	*p*-value	5-year LC, %	*p*-value
Total ^#^patients	26	42%		63%	
Age					
<16	12	42%	0.61	62%	0.96
≥16	14	42%		65%	
Sex					
Male	18	50%	0.33	67%	0.74
Female	8	25%		57%	
Performance status					
1	19	47%	0.65	63%	0.94
2	7	29%		64%	
Pathologic subtype					
Osteoblastic	13	61%	0.11	79%	0.09
Others	13	23%		45%	
Tumor location					
Pelvis	24	41%	0.84	61%	0.36
Others	2	50%		100%	
Tumor status					
Primary^a^	20	44%	0.48	61%	0.55
Others	6	33%		83%	
Tumor status					
Non-metastatic	23	43%	0.61	60%	0.33
Metastatic	3	33%		100%	
Target volume (cm^3^)					
<452	13	54%	0.14	81%	0.07
≥452	13	29%		38%	
Largest tumor diameter					
≤9.5 cm	14	64%	0.0013^c^	75%	0.03^c^
>9.5 cm	12	13%		45%	
Response to chemo					
SD + PR	12	48%	0.07	65%	0.29
Others^b^	11	18%		36%	
Total CIRT dose (Gy RBE)					
≥70.4	21	37%	0.72	55%	0.10
<70.4	5	60%		100%	

Abbreviations: ^#^number; OS, overall survival; LC, local control; SD, stable disease; PR, partial response; CIRT, carbon ion radiotherapy; Gy RBE, Gray Relative Biological Effectiveness.

^a^excluding locally recurrent (1) and radiation-induced (2) cases.

^b^Others include progressive disease and incomplete chemotherapy regimen; excluding unknown response.

^c^denotes statistical significance.

### Prognostic factors

In univariate analysis, only largest tumor diameter before starting CIRT was a significant prognostic factor for 5-year LC and OS (Table 3). Patients with smaller tumor diameter (≤9.5 vs. >9.5 cm) had improved 5-year OS (64 vs. 13%; *p* = 0.0013) and LC (75 vs. 45%; *p* = 0.03). Pathologic status (osteoblastic vs. others) and target volume (<452 vs. ≥452 cm^3^) trended towards significance for 5-year LC (*p*-values 0.09 and 0.07, respectively) whereas response to neoadjuvant chemotherapy (stable/partial response vs. others) trended towards significance for 5-year OS (*p* = 0.07).

### Adverse events and long-term complications

All patients tolerated and completed their CIRT treatments. There were no acute or late CIRT-related grade 5 toxicities. Two patients however died within 3 months after irradiation but the deaths were unrelated to CIRT. Thus, data was available for 24 patients for late toxicity analysis. Grade 3–4 adverse events excluding fractures of affected bone were observed in 4 cases. There was one case of grade 3 skin toxicity, one case of grade 4 skin toxicity, and 2 cases of neurologic dysfunction due to nerve injury. There was 1 case of grade 4 bone toxicity (Figure 1) in which the sacrum (S1) was involved with the disease and developed a fracture after CIRT. The patient underwent vertebral fixation surgery. However, the fixation device broke and had to be removed. After removing the hardware, the patient had difficulties with bowel movement and underwent a colostomy procedure. The patient is still alive without recurrence or metastasis and is able to ambulate using crutches.

There was no remarkable growth disturbance related to CIRT in any of the patients except those related to deformities caused by tumor or fractures. All patients (except one using wheelchair) were able to ambulate (with or without crutches or canes) after their treatment until recurrence, or death. Among the 9 patients surviving for >5 years, 3 patients were walking for their activities of daily living but using a wheelchair if walking for long distances. There were no cases of second malignant neoplasms after CIRT including the 10 patients who lived longer than 5 years (median 10 years; range 5–20.7 years).

## DISCUSSION

In this report, we present our 20-year experience using CIRT for pediatric and adolescent patients with inoperable osteosarcoma of the trunk, most of which are primary of the pelvis, with encouraging survival, maintenance of ambulation in most patients and acceptable adverse events. Although the most common primary bone malignancy in children, osteosarcoma is rare with an estimated age-adjusted incidence of 2–5 cases/million in the 0–24 years age group in the United States and world-wide [13, 14]. Moreover, truncal osteosarcomas represent only <5% of all cases and the pediatric truncal cases are even less [15]. Thus, it is difficult to design clinical trials or report results of pediatric osteosarcoma from single institutions given the heterogeneity in location, histology and treatment approaches. Despite the retrospective nature, the small number of patients and the relatively short follow-up in this study, this report is extremely valuable for the pediatric oncologists and the particle therapy community considering the treatment modality used, the rarity of the diagnosis and the encouraging results.

In multiple prior studies, truncal osteosarcomas had lower overall survival compared to extremity osteosarcomas due to the difficulty in performing adequate surgery. In the Cooperative Osteosarcoma Study (COSS) Group where patients received pre-operative and post-operative chemotherapy and surgery for operable lesions, overall survival in truncal osteosarcomas was 34% compared to 65% in all patients [2]. The majority of these patients underwent surgical resection. In a sub-analysis of the COSS cohort including only those with pelvic osteosarcoma, 17 out of 67 patients did not undergo any surgery. LC and 5-year OS were 30% and 27%, respectively for all patients compared to 6% and 0% in the 17 patients who did not undergo surgery (LC and 5-year OS for pelvic site in our study was 61% and 41%, respectively, Table 3) [16]. Likewise, similar poor survival of truncal osteosarcomas was found in other studies with a heterogeneous group of young or adult patients treated with combinations of surgery (for resectable cases), chemotherapy and radiation [3, 5, 15, 17]. To our knowledge, only one study separately reported the results of pediatric patients with pelvic osteosarcomas. Compared to 19 pediatric patients with pelvic osteosarcoma treated with combination of surgery (10 unresectable and 9 status post hemi-pelvectomy), chemotherapy and radiation, our study shows improved 5-year OS (41.7 vs. 26.3%) and DFS (34.6 vs. 15.8%) [18].

Proton therapy has also been used to treat truncal osteosarcomas. Two studies from the Massachusetts General Hospital (MGH) reported on the use of proton therapy in unresected or incompletely resected truncal osteosarcomas. The initial report included patients with spine (8 patients), pelvis (7), trunk (1), skull/face (17) or extremity (8) osteosarcomas who either were inoperable or received surgery with close or positive margins (65.85% with gross total resection, 21.95% subtotal resection, and 12.2% biopsy only). Because of the high risk of local recurrence after poor surgical techniques, all patients received RT and two-thirds received proton RT. Notably, overall survival was similar for gross or subtotal resection and was significantly lower in patients who only received biopsy [19]. In another report of unresected or partially resected osteosarcomas of the trunk (mostly) treated with proton or mixed proton/photon RT, overall survival and local control at 5 years were 67% and 72%, respectively [20]. However, only 36% of the patients had high-grade disease and only 22% did not receive any surgery at all. 35% and 44% of the cases received partial and gross resection (with positive margins), respectively. Moreover, the median target volume was 213 cm^3^ (compared to 452 cm^3^ in our study). Apparently, the baseline characteristics (resectability, grade and size) in this study are more favorable than ours. Indeed, tumor diameter was a prognostic factor predicting poor local control and overall survival, and target volume tended to significance for local control in our study (Table 3). Prior studies using CIRT for older patients with unresectable osteosarcoma also revealed that larger target volume was a prognostic factor for poor LC and OS [8]. Larger tumors have increased tumor heterogeneity and more hypoxia and thus increased resistance to treatment. While CIRT have low oxygen enhanced ratio (OER), it is not 1 across the width of the Spread out Bragg peak (SOBP) [21] which may contribute to some treatment failures. In addition, larger targets have larger areas of lower linear energy transfer (LET) in their center compared to smaller targets, which may be a risk factor for local failures. Moreover, patients with larger tumors like the ones seen in our cohort may be at an increased risk of distant metastasis which has a very grim prognosis. Many patients may have long-term local control but develop distant metastasis. It is unclear whether CIRT reduces distant metastasis since none of prior studies compared CIRT to other modalities in a randomized fashion. However, preclinical studies have shown that CIRT indeed reduces metastatic potential, cell migration, and invasion across different cell lines and in *in vivo* tumor models [22–24]. Our encouraging outcomes occurred while preserving ambulation in most patients and with few grade 3–4 adverse events. The 2 late skin toxicities (one grade 3 and one grade 4) occurred in 2 patients who received 2 port irradiation. It is possible that these toxicities could be avoided with 3–4 port irradiation.

Very few reports have been published on the use of CIRT in pediatric patients [25–28], all from the CIRT center in Heidelberg, Germany, with only a handful of osteosarcoma patients. In general, there is considerable reluctance to apply CIRT for pediatric patients primarily out of concern regarding treatment-related adverse events and second malignant neoplasms [29, 30]. While preclinical and modelling studies have reported conflicting results [31–34], the out-of-field dose from secondary neutrons is lowest for carbon ions delivered by scanning beams followed by protons delivered by scanning beams, then passive beams. Secondary neutrons dose was highest for high-energy photons [35, 36]. However, there has not been any epidemiologic studies of the second cancer risk after CIRT. Despite the short follow-up, no second cancers have been reported from the 3 pediatric studies from Germany and now we similarly report no cases of second malignancies after CIRT.

In conclusion, this is the first-ever series of inoperable truncal (mostly pelvic) pediatric osteosarcoma treated with CIRT and the results show that CIRT is safe and efficacious for these patients. Prospective studies and longer follow-up are needed before CIRT is standard for pediatric patients. Meanwhile, CIRT for pediatric osteosarcoma should be only done on a clinical trial where patients can be routinely followed for the long term especially that some late recurrences are expected.

## MATERIALS AND METHODS

### Patient characteristics

With the approval of our institutional review board (IRB # 16-018), we retrospectively reviewed medical records of all pediatric and adolescent patients who received CIRT at the Hospital of Charged Particles in the National Institute of Radiological Sciences (NIRS) between 1996 and 2014. This cohort of patients was included in 2 clinical trials for patients with sarcomas of any age group. Patients were required to have pathologically confirmed unresectable or medically inoperable high-grade osteosarcoma, with an Eastern Cooperative Oncology Group performance status of 0 to 2, without any other lesion at the time of CIRT, no prior conventional radiotherapy at the same site (excluding radiation-associated sarcomas), and no chemotherapy within 4 weeks of CIRT initiation. Patients who had an infection at the site of CIRT, or an intravascular tumor embolism were not allowed. Patients with tumors >15 cm were allowed to receive CIRT when the patch-field technique was available. All patients and/or their guardians signed informed consent prior to receiving CIRT.

### Carbon ion radiotherapy

In brief, carbon ion beams with 290 MeV/n, 350 MeV/n, and 400 MeV/n energies were generated by a synchrotron accelerator. These energies correspond to a range of water-equivalent depth of 15–25 cm. All patients were treated using passive irradiation. The tumor-specific spread-out Bragg peaks (SOBP) were modulated using wobbler magnets, beam scatterers, ridge filters, multi-leaf collimators, patient-specific collimators and compensation boluses. Patients were immobilized in the supine or prone position using thermoplastic shells and moldable cushions as needed depending on treatment site. Additional degrees of freedom were provided by our couch (–20° to +20°). Computed tomography (CT) images of 1–5 mm thickness were acquired for treatment planning which was done using the HIPLAN software (NIRS, Chiba, Japan). The clinical target volume (CTV) included the gross tumor volume (GTV) and all areas suspicious for microscopic involvement as previously described [8]. The planning target volume (PTV) included the CTV with an additional 1–5 mm margin depending on the target shape, size and the selection of collimators. Irradiation was performed using 3 ports for the majority of patients (range 2–6). One port was used per fraction, for a total of 16 fractions, delivered in 4 weeks, with 4 fractions per week. Dose is expressed as Gray Relative Biological Effectiveness (Gy RBE) which is the carbon physical dose x RBE. Dose prescription was based on prior clinical trials of sarcoma at the NIRS as there were no clinical trials specific for patients in the pediatric age group [8, 11, 37]. The CIRT irradiation system have been previously described [38, 39].

### Evaluation of patient outcomes

Patients were routinely evaluated via physical examinations and imaging studies (CT or MRI) every 3–6 months until death or until date of being lost to follow-up. For patients who lived far from our hospital and could not travel routinely, we acquired their imaging and follow-up details from their primary physicians. The follow-up period was counted from CIRT start date. Response to chemotherapy was evaluated using the RECIST criteria [40]. Late adverse events were evaluated using the National Cancer Institute Common Toxicity Criteria, version 3.0. Local control (LC) was calculated from date of CIRT initiation till date of local failure (LF). Locally controlled patients were censored at last follow-up or death. Progression-free survival (PFS) was calculated from date of CIRT initiation till date of LF, distant metastasis (DM) or death. Overall survival was calculated from date of CIRT initiation till date of death. Alive patients were censored at time of last follow-up.

### Statistics

LC, PFS and OS were plotted using the Kaplan-Meier method (GraphPad, La Jolla, California). Univariate analysis was done using the log-rank test. All statistical tests were two-sided and a *p*-value of ≤ 0.05 was considered statistically significant for all comparisons.
